# High-resolution analysis of HLA class I alterations in colorectal cancer

**DOI:** 10.1186/1471-2407-6-233

**Published:** 2006-10-02

**Authors:** Jan Willem F Dierssen, Noel FCC de Miranda, Arend Mulder, Marjo van Puijenbroek, Willem Verduyn, Frans HJ Claas, Cornelis JH van de Velde, Gert Jan Fleuren, Cees J Cornelisse, Willem E Corver, Hans Morreau

**Affiliations:** 1 Department of Pathology, Leiden University Medical Center, Leiden, The Netherlands; 2 Department of Immunohemathology and Blood Transfusion, Leiden University Medical Center, Leiden, The Netherlands; 3 Department of Surgery, Leiden University Medical Center, Leiden, The Netherlands

## Abstract

**Background:**

Previous studies indicate that alterations in Human Leukocyte Antigen (HLA) class I expression are frequent in colorectal tumors. This would suggest serious limitations for immunotherapy-based strategies involving T-cell recognition. Distinct patterns of HLA surface expression might conceal different immune escape mechanisms employed by the tumors and are worth further study.

**Method:**

We applied four-color multiparameter flow cytometry (FCM), using a large panel of alloantigen-specific anti-HLA-A and -B monoclonal antibodies, to study membranous expression of individual HLA alleles in freshly isolated colorectal cancer cell suspensions from 21 patients.

**Results:**

Alterations in HLA class I phenotype were observed in 8 (38%) of the 21 tumors and comprised loss of a single A or B alleles in 4 cases, and loss of all four A and B alleles in the other 4 cases. Seven of these 8 tumors were located on the right side of the colon, and those showing loss of both HLA-A and -B membranous expression were all of the MSI-H phenotype.

**Conclusion:**

FCM allows the discrimination of complex phenotypes related to the expression of HLA class I. The different patterns of HLA class I expression might underlie different tumor behavior and influence the success rate of immunotherapy.

## Background

The high morbidity and mortality of colorectal cancer underscores the need for new, more effective adjuvant treatment strategies. One promising approach centers on boosting cell-mediated anti-tumor immune responses. Numerous tumor-associated peptides have been identified that can be specifically targeted with vaccine-based immune therapy strategies [1]. Specifically, such strategies may be employed for colorectal tumors with a so-called microsatellite instability-high (MSI-H) phenotype [2,3]. These tumors are characterized by a deficient DNA mismatch repair mechanism which results in an abundance of frame shift mutations of (coding) microsatellite repeat sequences. The peptides produced by such frame shift mutations can evoke specific anti-tumor immune responses [4-6]. Nevertheless, however promising the approach, its success is dependent on the extent of operational immune surveillance mechanisms in the tumor.

Human Leukocyte Antigen (HLA) class I molecules are of major importance for cell-mediated anti-tumor immune responses. Expression of HLA class I/β2-microglobulin (β2-m) complexes carrying tumor-specific peptides is a prerequisite for adaptively matured cytotoxic T cells (CTLs) to be able to recognize tumor cells [7]. HLA class I antigens are encoded by a family of highly polymorphic genes, with each allele responsible for a different repertoire of antigen presentation. Thus, even the loss of a single allele could potentially allow the escape from an antigen-specific anti-tumor response. Loss of expression of HLA class I molecules has been frequently reported for colorectal tumors [8-10]. This would therefore represent a serious limitation for vaccine-based anti-tumor therapies.

However, these studies have primarily been based on immunohistochemical analyses (IHC) and therefore have a number of intrinsic limitations [11]. HLA staining by IHC is often strongly cytoplasmic, which could potentially obscure functionally relevant membranous co-expression and result in a false negative interpretation. Additionally, adequate study by IHC is hampered by the limited choice of antibodies (Abs) available for the analysis of formalin-fixed, paraffin-embedded tissue and importantly, despite the fact that the use of fresh frozen tissue allows the employment of a higher number of Abs, complete panels of the latter are not yet readily available. Another important consideration is that certain HLA class I complexes also mediate inhibitory signals through receptors expressed by CTLs and natural killer (NK) cells, averting the so-called 'missing self' recognition [12,13]. Hence, an effective tumor immune escape mechanism could occur through a subtle alteration of the tumor cell HLA phenotype, circumventing both CTL and NK cell attack. Thus far, it has not been technically feasible to study these intricacies comprehensively, and the molecular mechanisms proposed to generate HLA alterations have been contradictory, as they could not always account for the observed phenotypic alterations.

In an attempt to overcome these limitations, we have employed four-color multiparameter flow cytometry (FCM) on freshly isolated tumor cells using a large panel of human alloantigen specific monoclonal antibodies (mAbs). This approach has numerous advantages: i) it allows for the study of complex phenotypes, ii) FCM is much more sensitive than IHC, and iii) it is possible to study membranous HLA expression alone. Using the FCM technique we were able to distinguish two distinct patterns of altered HLA expression restricted to specific colorectal tumor subsets.

## Methods

### Patient material

Fresh tumor samples, macroscopically identified by a pathologist (HM), were collected from 21 patients with surgical resections between 2001 and 2003 at the department of Surgery of the Leiden University Medical Center. Peripheral blood samples were collected from 15 patients pre-operatively. There was no pre-selection of the patients included in this series. All patients agreed to participate and provided written informed consent, and the study was approved by the local ethical review committee.

### HLA genotyping

DNA was extracted from peripheral blood leucocytes using a standard salting-out procedure. Patients were HLA-A and -B genotyped using PCR-sequence-specific oligonucleotide probes (Dynal Biotech Ltd., Wirral, U.K.), as described previously [14]. Patient HLA genotype is shown on Table 2.

### Tissue dissociation

Minced fresh tumor samples were weighed, cut in fragments of approximately 1 mm^3^, and incubated over night at 4°C with 5 ml/g serum-free RPMI 1640 medium (Invitrogen, Paisley, UK) supplemented with 50 IU/ml penicillin-streptomycin (ICN Biomedicals Inc., Aurora), 128 U/ml collagenase type I (Sigma-Aldrich, St. Louis, MO), 100 U/ml hyaluronidase type V Sigma-Aldrich), and 250 U/ml DNAse I (Sigma-Aldrich). The next day, the same suspension was incubated for 90 min at 37°C followed by two washing steps with RPMI including 10% FCS (Invitrogen) to block proteolysis, and Hank's Balanced Salt Solution (Sigma-Aldrich). Cells were incubated for another 15 min at 37°C with 0.25% Trypsin (Invitrogen), 1 mM EDTA in Hank's Balanced Salt Solution, chilled to 4°C, and washed with RPMI with 10% FCS. Finally, the suspension was filtered through a 100 μm sieve (Verseidag-Industrietextilien GmbH, Kempen, Germany) and used immediately for flow cytometry.

### Anti-HLA antibodies

A large panel of human and mouse anti-HLA mAbs was used for FCM (Table 1), some of which were previously described [15-17]. To establish the activity of the human mAbs (hu-mAb), peripheral blood lymphocytes were isolated from multiparous women who had developed HLA alloantigen specific antibodies during pregnancy, and the lymphocytes transformed with Epstein Barr virus (EBV). HLA antibody producing EBV cell lines were stabilized by electrofusion and rigorous cloning [18]. The specificity of hu-mAbs was determined by testing these antibodies with a large (n > 240) panel of HLA-typed peripheral blood mononuclear cell suspensions in a conventional complement dependent microcytotoxicity (CDC) assay [19]. On the basis of the mAb reactivity patterns, suitable HLA alloantigen-specific sub-panels were chosen for each colorectal cancer patient's HLA phenotype, as well as proper negative isotype controls. Several mAbs were used to address the same HLA allelic products to rule out artifacts caused by differences in mAb in specificity/affinity.

### Flow cytometry

A seven-step, four-color staining procedure was performed as described previously [20]. Simultaneous labeling of HLA, β2m, DNA, and the intermediate filaments keratin and vimentin enabled us to discriminate HLA expression in keratin positive (tumor) epithelial cells from those in vimentin positive 'normal' cells (leucocytes and fibroblasts). Importantly, we specifically analyzed membranous expression since cells were only permeabilized after HLA immunostaining making the cytoplasm inaccessible to the anti-HLA Abs. In 15 cases, we used a complete panel of human and mouse anti-HLA mAbs, and in another 5 cases we used mouse mAbs (mu-mAb) because the HLA genotype was not known prior to tumor resection. Reagents used were biotinylated goat anti-mouse and anti-human mAbs (Southern Biotechnology Associates, Birmingham, AL), Streptavidin-allophycocyanin (APC) (Molecular Probes, Eugene, OR), goat F(ab')_2 _anti-mouse IgG1 and IgG2a-RPE, and goat F(ab')_2 _anti-mouse IgG2b or IgG1-FITC polyclonal antibodies (SBA), paraformaldehyde (Merck, Whitehouse Station, NJ), L-α-lysophosphatidylcholine (lysolecithin, Sigma-Aldrich), RNase (Sigma-Aldrich), and propidium iodide (Calbiochem) were used. We used three different combinations of anti-keratin and anti-vimentin mAbs to prevent cross-reactivity of the secondary reagents with the anti-HLA antibody. The first combined anti-keratin IgG1 mAbs M9, M20, and AE1/AE3 (Chemicon International Inc., Temecula, CA) and anti-vimentin Ig2b mAb V9; the second combined anti-keratin IgG2a mAbs CAM5.2 (BD Biosciences, San Jose, CA) and anti-vimentin IgG1 mAb V9; and the third combined anti-keratin IgG2a mAb CAM5.2 and anti-vimentin Ig2b mAb V9. Clones M9, M20, and V9 were established at the Department of Pathology, Leiden University Medical Center, The Netherlands, and are commercially available from ARA, Alphen a/d Rijn, the Netherlands. For each sample, measurements from 10,000–20,000 single cells were collected using a standard FACSCalibur™ flow cytometer (BD Biosciences). Data were analyzed using WinList 5.0 and ModFit 3.0 software (Verity Software House Inc., Topsham, ME). After electronic gating on keratin and vimentin content, single parameter histograms were obtained for DNA content and HLA expression. Geomean APC fluorescence intensity values (MFI) were subtracted by MFI values from proper negative controls. These ratios were used to calculate the relative expression value (REV) of keratin positive (ker+) cells compared to vimentin positive (vim+) cells.

REV=MFI[ker⁡+;HLA]−MFI[ker⁡+;negative control]MFI[vim+;HLA]−MFI[vim+;negative control]
 MathType@MTEF@5@5@+=feaafiart1ev1aaatCvAUfKttLearuWrP9MDH5MBPbIqV92AaeXatLxBI9gBaebbnrfifHhDYfgasaacH8akY=wiFfYdH8Gipec8Eeeu0xXdbba9frFj0=OqFfea0dXdd9vqai=hGuQ8kuc9pgc9s8qqaq=dirpe0xb9q8qiLsFr0=vr0=vr0dc8meaabaqaciaacaGaaeqabaqabeGadaaakeaacqqGsbGucqqGfbqrcqqGwbGvcqGH9aqpdaWcaaqaaiabb2eanjabbAeagjabbMeajjabcUfaBjGbcUgaRjabcwgaLjabckhaYjabgUcaRiabcUda7iabbIeaijabbYeamjabbgeabjabc2faDjabgkHiTiabb2eanjabbAeagjabbMeajjabcUfaBjGbcUgaRjabcwgaLjabckhaYjabgUcaRiabcUda7iabb6gaUjabbwgaLjabbEgaNjabbggaHjabbsha0jabbMgaPjabbAha2jabbwgaLjabbccaGiabbogaJjabb+gaVjabb6gaUjabbsha0jabbkhaYjabb+gaVjabbYgaSjabc2faDbqaaiabb2eanjabbAeagjabbMeajjabcUfaBjabbAha2jabbMgaPjabb2gaTjabgUcaRiabcUda7iabbIeaijabbYeamjabbgeabjabc2faDjabgkHiTiabb2eanjabbAeagjabbMeajjabcUfaBjabbAha2jabbMgaPjabb2gaTjabgUcaRiabcUda7iabb6gaUjabbwgaLjabbEgaNjabbggaHjabbsha0jabbMgaPjabbAha2jabbwgaLjabbccaGiabbogaJjabb+gaVjabb6gaUjabbsha0jabbkhaYjabb+gaVjabbYgaSjabc2faDbaaaaa@9383@

### Immunohistochemistry

A standard three-step, indirect immunohistochemistry was performed on consecutive 4 μm formalin-fixed, paraffin-embedded tissue sections mounted on aminopropylethoxysilane coated glass slides. Antigen retrieval was performed in boiling citrate 10 mM (pH 6.0) for 20 min followed by a peroxidase block with 0.03% hydrogen-peroxide methanol and an endogenous avidin-binding activity-block by incubation with avidin solution for 15 min (one chicken egg white resuspended in 100 ml PBS) and 15 min in biotin solution (pasteurized cow milk), and di-aminobenzidine development. Antibodies used included biotinylated rabbit anti-mouse (DAKOCytomation, Glostrup, Denmark), goat anti-rabbit (DAKOCytomation), biotinylated-peroxidase streptavidin complex (SABC; DAKOCytomation), anti-HLA-A heavy chain mAb HCA2, anti-HLA-B/C heavy chain mAb HC10 (supernatant kindly provided by Dr. J. Neefjes, NKI, Amsterdam, The Netherlands and Dr. H. L. Ploegh, MIT, MA, at 1:400 and 1:100 dilutions, respectively) [21,22], rabbit anti-β2-m polyclonal Ab (A 072; DAKOCytomation; 167 μg/l), anti-MLH1 (clone G168-728; BD Biosciences Pharmingen; 20 mg/l), and anti-PMS2 (clone A16-4; BD Biosciences PharMingen, San Diego, CA, USA; 10 mg/l). Loss of expression was defined as complete loss of staining in both the membrane and cytoplasm (HCA2, HC10, and anti-β2-m) or in the nucleus (anti-MLH1 and anti-PMS2) with concurrent expression in normal epithelium, stroma, or infiltrating leukocytes.

### Microsatellite instability analysis

Microsatellite instability (MSI) analysis was performed and scored as described previously [23] using the markers BAT25, BAT26, BAT40, D2S123, D5S346, D17S250, MSH3 and MSH6. *β2m *frameshift analysis was performed with primers (available upon request) build around coding repeats of the *β*_2_*m *gene; the (CT)4 repeat in exon 1 and two (A)5 repeats in exon 2[24]. Genomic DNA of both normal and tumor tissue was isolated from 3 tissue cores of 0.6 mm diameter of formalin-fixed, paraffin-embedded material using the Chelex extraction method [25].

### Statistics

Values of significance were calculated using the software package SPSS 10.0.7 (SPSS Inc., Chicago, IL, USA). We performed Pearson Chi-square tests or, when expected count was below 5, Fisher's exact 2-sided tests. The independent sample t-test was performed to test equality of means of the age at operation.

## Results

### Two patterns of alteration in HLA class I phenotype as observed by flow cytometry

**Figure 1:**
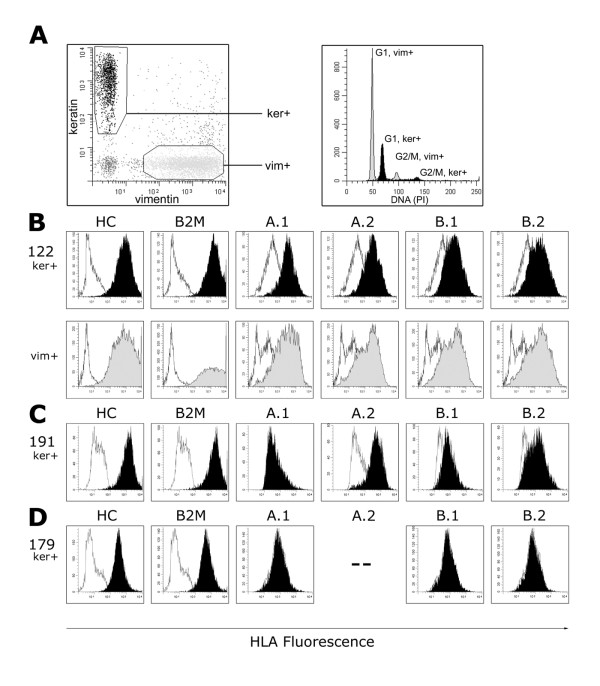
Membranous HLA class I expression analysis by four-color multiparameter flow cytometry of colorectal tumor cell suspensions. Fluorescence intensities of HLA class I with antibodies W6/32 (HC), BBM.1 (B2M), and alloantigen-specific antibodies against single A and B alleles (A.1 – B.2; see Table 2) are displayed in filled curves; corresponding negative controls are in non-filled curves. A, keratin positive (ker+) epithelial cells are distinguished from vimentin positive (vim+) stroma cells and infiltrating leukocytes. DNA ploidy analysis of case 122 reveals an aneuploid ker+ tumor cell fraction (black) compared to the normal diploid vim+ cells (light gray). B, Expression of all HLA molecules tested in case 122 of both ker+, tumor (black), and vim+ 'normal' (gray) cells. C, loss of the single A.1 (HLA-A2) allele was observed for tumor cells in case 191. D, loss of expression of all 4 HLA-A and -B alleles in tumor cells, but retention of HC and B2M, in case 179. The patient is homozygous for HLA-A3 (--;see Table 2).

Loss of expression of HLA class I alleles was observed in 8 of 21 (38%) colorectal cancer cases studied [see Additional file 1]. Two distinct patterns of HLA loss were identified; the loss of a single HLA-A or -B allele which was observed in 4 of 8 cases (cases 61, 63, 109, and 191), and the loss of both HLA-A and -B alleles which was observed in the remaining 4 cases (cases 55, 56, 120, and 179). Importantly, in the latter group, membranous expression of β2-microglobulin (β2-M) and remaining HLA class I antigens was retained in the 4 tumors, but was diminished as determined with the mAbs BBM.1 and W6/32. In these samples, the average Relative Expression Values (REV, see Methods section) was 0.43 and 0.38, respectively, compared to the average REVS of 2.9 and 2.2, respectively, in the other 17 cases (Figure 1) [see also Additional file 1]. This would suggest the retention of at least one of the other HLA class I alleles, i.e. HLA-C, -E, -F or -G.

### Alterations of HLA phenotype are found in specific subsets of colorectal cancer

The 7 of 8 cases with HLA alterations were all right sided (p = 0.024). Interestingly, all 5 MSI-H cases in the series (5/21) demonstrated HLA alterations [see Additional file 1]. Four of the 5 cases demonstrated loss of HLA-A and -B (p = 0.028); in 1 of the 5 cases, we observed the loss of a single HLA allele (case 191). Loss of expression of the heterodimer of MLH1 and PMS2 was observed in the 4 MSI-H cases through IHC, and was not observed in any other cases [see Additional file 1]. These patients do not fulfill any criteria indicative of HNPCC are thus most likely sporadic. Other clinicopathological features typical of sporadic MSI-H tumors [26] were also present; they occurred in elderly patients (mean age 75 years, p = 0.043), 3 of 4 were located in the caecum (p = 0.019), and 3 of 4 were peri-diploid (p = 0.053). The other MSI-H tumor (case 120) was located in the sigmoid, occurring in the setting of the Hereditary Non-Polyposis Colorectal Cancer (HNPCC) syndrome. A mutation in the *Mut-S-Homologue 2 *(*MSH2*) gene (G674R, exon 13 2020G>C) segregated in this particular family, a variant that is probably pathogenic. The abrogation of MSH2 expression in this tumor was confirmed by IHC. Since all the 4 MSI-H tumors that lost HLA A and B expression showed diminished membranous expression of β2m we have screened the microsatellite sequences of the corresponding gene but failed to find any frameshift mutation.

**Figure 2:**
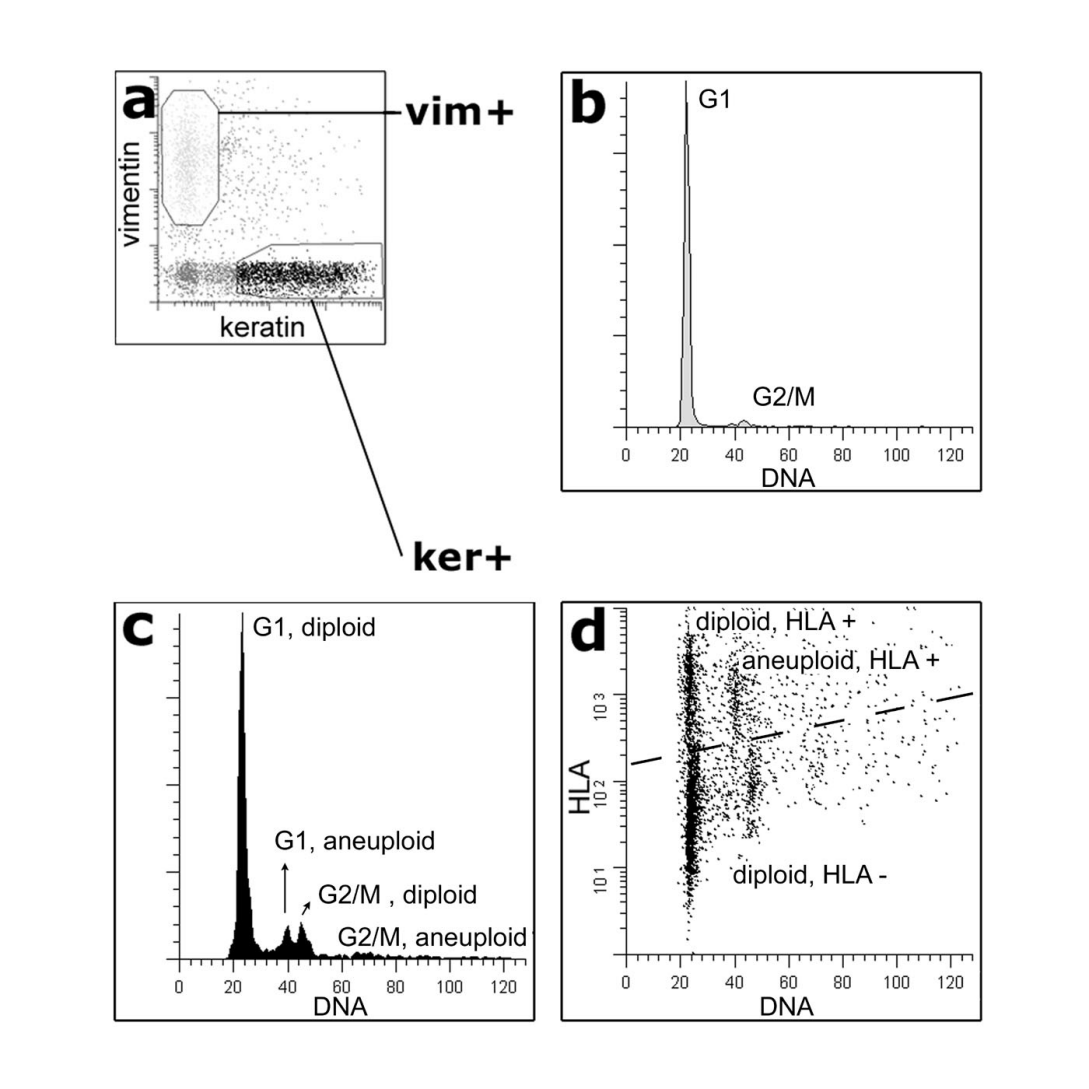
HLA phenotype alteration is associated with DNA ploidy of the cells within one tumor. Flow cytometry analysis of case 55 reveals a synchronous divergence of tumor cell populations with DNA ploidy and HLA expression. Keratin positive (ker+, black) epithelial cells are distinguished from vimentin positive (vim+, light gray) stroma cells and infiltrating leukocytes (a). Compared to the diploid vim+ cells (b), peri-diploid and aneuploid cell populations of ker+ cells are revealed (c). By plotting HLA expression to DNA content, 3 ker+ cell populations can be discriminated, including a HLA negative DNA diploid tumor cell population and a HLA positive aneuploid population (d).

One case (case 55, MSI-H, multi-ploid) was particularly intriguing. Ploidy studies indicated a peri-diploid tumor cell population as well as an aneuploid population (Figure 2). Interestingly, while the aneuploid population retained HLA expression for all HLA alleles examined, the diploid population was characterized by two populations of cells; one population that retained HLA expression, and one population (the largest tumor cell population) that demonstrated a loss of expression of both HLA-A and -B alleles.

The 4 cases showing loss of both HLA-A and -B expression have not presented with lymph node or distant metastases to date, while 3 of the 4 cases with loss of a single allele (including 1 MSI-H case) and 10 of the remaining 13 cases without alterations of the HLA phenotype, did metastasize (p = 0.035) to either lymph nodes or to distant extranodal sites.

### Comparison of FCM with conventional immunohistochemistry

Using FCM, we detected a significantly lower frequency of HLA class I aberrations than previously reported [8-10,27]. Consequently, we decided to compare our FCM results with those obtained using conventional IHC. The human anti-HLA mAbs used for flow cytometry could clearly not be used for the IHC analyses due to high background staining caused by non-informative cross-reactivity of the secondary anti-human antibody. Consequently, we chose to use a panel of mouse anti-HLA antibodies (HCA2, HC10, anti-β2-m) for the IHC analyses, although these are not alloantigen specific. Loss of expression of a single HLA allele could not be detected using these antibodies.

**Figure 3:**
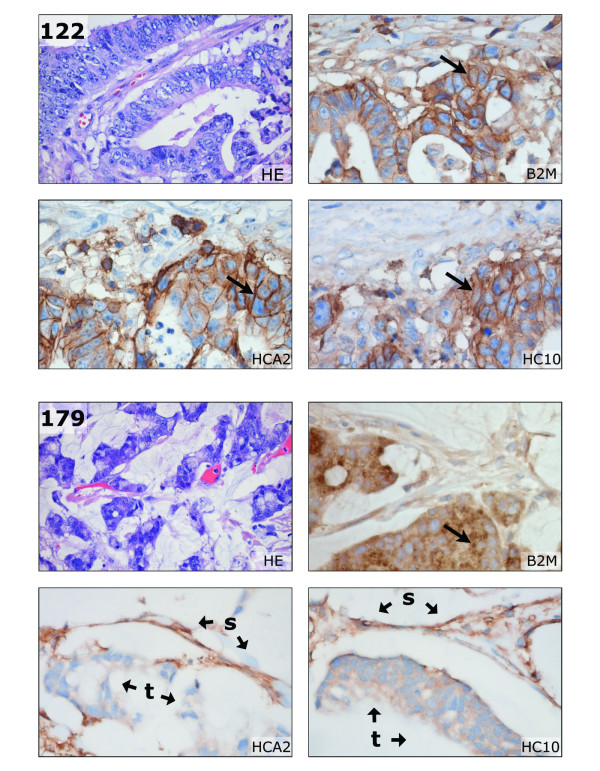
Conventional HLA immunohistochemistry of formalin-fixed paraffin-embedded tissue. Using HCA2 (HLA-A heavy chain), HC10 (HLA-B/C heavy chain) and anti-B2M antibodies, loss of HLA A and B expression as detected by flow cytometry (see Figures 1 and 2) could be confirmed. In the top panel, membranous staining (arrows) of B2M, HCA2, and HC10 is observed in the moderately differentiated sigmoid adenocarcinoma of case 122. In the bottom panel, loss of HLA-A and -B is illustrated for the mucinous caecum adenocarcinoma of case 179. Tumor B2M staining was mainly restricted to the cytoplasm (arrow), but some membranous expression of B2M was observed by FCM (see Figure 1). The HCA2 and HC10 staining was completely lost in tumor epithelium (t). Typically, the retained HLA expression of stroma cells (s) resulted in an inverted staining pattern in comparison to case 122. HE, haematoxylin and eosin staining. Pictures were made at 400 × magnification, HEs at 100×.

IHC of cases 55, 56, 120, and 179 (the 4 HLA-A and -B negative cases) showed complete absence of HCA2 and HC10 staining in tumor cells, and decreased anti-β2-m staining which was primarily cytoplasmic (see Figure 3) and, consequently, probably irrelevant since cytoplasmic localization of HLA molecules is not functional. However, membranous staining of HCA2, HC10, and anti-β2-m was clearly observed in the adjacent stroma cells (positive internal control). The FCM data would thus appear to corroborate the IHC findings. Loss of staining was not observed in the remaining cases studied by IHC [see Additional file 1]. A heterogeneous staining pattern, i.e. both positively and negatively staining tumor fields within the same section, was frequently observed; such heterogeneity in membranous expression was not observed as discrete populations (peaks) in our FCM data.

## Discussion

We used four-color multiparameter FCM to study complete HLA-A and -B phenotypes in colorectal tumors using a large panel of alloantigen-specific mAbs. FCM allows the detection of variation in expression over a dynamic range of 4 logarithmic decades and therefore FCM appears to be very sensitive. Loss of membranous expression was detected in 38% (8 of 21 cases) of tumors in our study. Previous studies based on IHC demonstrated variable alterations in up to 73% of cases [8-10,27]. This discrepancy may be explained by the different technical approaches used and/or by the different composition of the groups under study (e.g. microsatellite stable/MSI-H distribution, tumor staging).

Except for one case (case 55) our FCM data did not provide evidence for discrete intratumoral epithelial subpopulations of differing HLA expression as was observed using IHC, although the lognormal distribution of the measured fluorescence may obscure these variations in expression. Nevertheless, the heterogeneous IHC staining patterns are difficult to interpret, and may be falsely regarded as loss of expression.

Using FCM, we characterized two distinctive alterations in colorectal tumors. We found 4 cases demonstrating loss of a single HLA-A or B allele (alterations not identified through immunohistochemistry), and 4 cases demonstrating loss of expression of both the A and B alleles, but not loss of all HLA class I molecules. The expression of HLA I antigen complexes is a prerequisite for the optimal targeting of tumor cells by CD8+ CTLs. However, NK cells and CTLs also carry inhibitory receptors for specific HLA class I alleles. Immunoselection thus relies on a delicate balance between HLA allele loss and retention. Examples of tumor immune escape due to such intricate alterations of HLA phenotype have been described recently [16,28]. In tumors showing loss of a single allele in our study, this loss was restricted to HLA-A2 in 3 of the 4 cases. HLA-A2 is not a ligand for inhibitory receptors. Those tumors may have effectively shed (HLA-A2) restricted epitopes and remained inhibitory to NK cell attack. Additionally, the 4 cases demonstrating loss of both HLA-A and -B alleles (also applicable to the cases showing loss of a single allele) retained expression of some total HLA class I molecules, which was detected through the W6/32 antibody and might indicate retention of HLA-C or other non classical HLA molecules like HLA-G or -E.. Of these, HLA-E is normally expressed in colon epithelial cells [29] and predominantly function as inhibitors of NK-cell function [30]. These tumor cells may escape CTL attack and attack by a subset of NK cells. HIV-1 infected cells, for example, avoid both CTL and NK cell-mediated lysis through specific HLA-A and -B downregulation caused by the HIV-1 gene product nef [31]. These different phenotypic alterations may reflect differences in local immune selective forces between the tumor subsets. Such differences remain to be identified.

All but one of the tumors with an altered HLA phenotype were located on the right side of the colon. The exception was a left sided tumor arising in the setting of HNPCC. Furthermore, the loss of both HLA-A and -B alleles was found exclusively in tumors with the MSI-H phenotype. Mismatch repair deficiency has previously been associated with frequent mutations in the *β2-m *gene (*B2M*)[24,32-35]. This gene harbors multiple short tandem repeats that are preferentially mutated, leading to total loss of membranous HLA class I expression. However, we did not observe frame shift mutations in these repeats in the MSI-H tumors with HLA-A and -B loss, which is in line with the retention of membranous staining (although diminished) with the W6/32 and anti-β2-m mAbs. Yet, the common loss of expression of HLA-A and -B in 3 of 4 sporadic MSI-H tumors, which is not due to *B2M *frame shift mutations, would suggest that another general mechanism may cause this altered phenotype.

Sporadic MSI-H tumors are usually relatively large, but rarely disseminate [3,36,37]. This favorable tumor behavior has been associated with an increased intra-epithelial CD8+ CTLs and CD57+ NK cells infiltrate when compared with the microsatellite stable tumors. CD4+ cells are not frequently found in the intraepithelial infiltrate of MSI-H tumors. [24,38-42]. The MSI of numerous coding microsatellites results in a large repertoire of immunogenic frame shift peptides which can give rise to specific anti-tumor immune responses [4-6]. However, these infiltrating lymphocytes may lead to the selection of HLA alterations in tumor cells. High levels of infiltrate were present in the HLA-A and -B negative cases in this study as observed by microscopy. Alternatively, the observed T cell infiltrate could represent an innocent bystander effect. Previously, we identified loss of HLA-A and -B expression in 6 of 88 cases of sporadic colorectal cancer using IHC that, surprisingly, did not present with recurrences or metastases during follow-up [27]. Although we could not determine the exact HLA phenotypic alteration at that time, the previous cases resemble the 3 sporadic MSI-H cases found in this study that lacked HLA-A and -B expression and that have not presented with lymph node or distant metastases to date. The NK cells that reside in the lymph and blood and that specifically kill metastasizing tumor cells (that lack total HLA class I expression) may explain the more favorable prognosis. However, the postulated expression of the remaining non HLA-A and -B alleles by tumor cells may specifically inhibit these NK cells.

## Conclusion

FCM allows the discrimination of complex phenotypes related to the expression of HLA class I. The different patterns of HLA class I expression might underlie different tumor behavior and influence the success rate of immunotherapy.

## Competing interests

The author(s) declare that they have no competing interests.

## Authors' contributions

JWD – Performed the tumor cell isolation, the flow cytometry procedure, carried out the immunohistochemistry assays, and performed the statistical analysis as well as being involved in drafting the manuscript.

NM – Involved in the interpretation of the data, drafting of the manuscript, and critical revision.

AM – Supervised the production of the HLA allele specific human monoclonal antibodies.

MP – Performed the microsatellite instability analysis.

WV – Performed the HLA genotyping

FC – Supervised HLA genotyping

CV – Surgeon responsible for the resections of colorectal samples used in the study

GJF – Contributed to the conception and design of the study, and to critical revision of the manuscript

CC – Contributed to the conception and design of the study, and critically reviewed the manuscript

WC – Supervised the technical procedures that involved flow cytometry and the acquisition of data.

HM – Contributed to the conception and design of the study, and responsible for study

All authors read and approved the final manuscript.

## Pre-publication history

The pre-publication history for this paper can be accessed here:



## Supplementary Material

Additional File 1 HLA phenotype alterations and clinicopathological features of colorectal tumors and their mismatch repair status. These data provides the HLA phenotypes assessed by flow cytometry of the different tumors and their clinicopathological features and mismatch repair status. **Clinicopathology**: loc., tumor localization: A, colon ascendens; Ce, caecum; D, colon descendens; R, rectum; RS, rectosigmoid; S, sigmoid; modified Dukes stages [43]; F-U, follow-up (max. 2 years): DOD, dead of disease; DND, dead but not due to disease; M, distant metastasis; nr, no recurrences. **MMR**, mismatch repair status: MSI, microsatellite instability: H, MSI-High; S, microsatellite stable; *, HNPCC, hereditary non-polyposis colorectal cancer; +, tumor epithelium staining positive as described in text; -, no staining of tumor cells, but staining of 'normal' cells, - +, heterogeneous staining of tumor cells; **FCM**, flow cytometry: A, aneuploid; D, diploid; M, multiploid; **HLA FCM**: Relative HLA Expression Values were calculated from flow cytometry analyses as described in the methods section. Depicted in black are cases in which fluorescence intensity of ker+ cells was equal to the negative control (see Figure 1). A.1 – B.2, HLA-A and -B alleles as depicted in Table 2; HC, HLA heavy chain expression detected with the W6/32 antibody. †, HLA-A and -B negative and positive populations are present, therefore REV is not informative (see Figure 2); ‡, homozygous HLA-A genotype. **HLA IHC**, immunohistochemistry of HLA molecules: +, tumor epithelium staining positive as described in text; -, no staining of tumor cells, but staining of 'normal' cells; c, tumor epithelium staining restricted to the cytoplasm; -+, heterogeneous staining of tumor epithelium. Click here for file

## Figures and Tables

**Table 1:** Monoclonal anti-human antibodies used for flow cytometry analysis of HLA Class I expression

mAb	species	isotype	HLA specificity	*reference*
116.5.28	M	IgG2a	Bw4	*
126/39	M	IgG3	Bw6	*
A11.1M	M	IgG3	A11, A24	*18*
BB7.2	M	IgG2b	A2, Aw68	*18*
Bbm1	M	IgG2b	β 2-m	*18*
GAP.A3	M	IgG2a	A3	*18*
MA2.1	M	IgG1	A2, B17	*18*
SFR8.B6	R	IgG2b	Bw6	*18*
W6/32	M	IgG2a	HLA -A,-B,-C	*18*
BV 8E9	H	IgM,κ	A28, A33, A34	*16*
BVK 5B10	H	IgM,κ	B8	*†*
BVK 5C4	H	IgM,κ	A80, A9	*21*
DMS 4G2	H	IgG1,λ	B62, B35	*17*
FVS 4G4	H	IgM,κ	B15, B17, B5, B37, B16, B18, B35	*16*
GK 31F12	H	IgM,κ	B13	*16*
GV 5D1	H	IgG1,λ	A1, A9	*16*
GVK 10H7	H	IgM,λ	B5, B35, B18, B37, B38, B14, B77, B72, B53	*†*
GVK 4H11	H	IgM,κ	B35, B62, B5, B16, B18, B37, B53, B70, B14	*†*
HDG 2G7	H	IgG1,κ	A19, B17, B63, B47	*16*
HDG 8D9	H	IgG1,λ	B51, B35	*16*
IN 2D12	H	IgM,λ	B15, B35, B21, B70	*16*
JOK 3H5	H	IgM,λ	B40, B21, B13, B12, B41, B70	*†*
KAL 3D5	H	IgG1,λ	B51, B52, B77	*17*
KG 30A7	H	IgM,λ	B27, B12, B14, B49	*18*
KLL 5E10	H	IgG1,κ	B51, B52	*†*
MUS 4H4	H	IgG1,λ	Bw4	*†*
Nie 44B8	H	IgM,κ	A10	*16*
OK 1C9	H	IgM,λ	A3, A11, A33, A31, A26	*18*
OK 2F3	H	IgM,κ	A3	*16*
OK 2H12	H	IgM,κ	A11, A3, A36, A32, A1	*16*
OK 3C8	H	IgM,κ	A3, A11, A32, A36, A31	*16*
OK 4F10	H	IgM,κ	A1, A3, A11, A31, A33, A26, A29, A30	*†*
OK 4F9	H	IgM,κ	A1, A36, A3, A11, A34, A66, A26, A29, A30, A31, A33	*16*
OK 5A3	H	IgM,λ	A1, A3, A11, A24, A36	*18*
OK 6H10	H	IgM,κ	B15, B21, B56, B35, B72	*16*
OK 6H12	H	IgM,κ	B21, B56, B70, B35, B62	*18*
ROU 9A6	H	IgG3,λ	B12, B13, B40, B21, B41	*†*
SN 607D8	H	IgG1,κ	A2, A28	*16*
SN 66E3	H	IgM,κ	A2, A28	*16*
vD1F11	H	IgM,λ	B62, B35, B57, B21, B56, B70, B55	*16*
VTM 1F11	H	IgG1,κ	B27, B7, B60	*†*
VTM 4D9	H	IgG1,κ	B7, B27	*†*
VTM 9A10	H	IgG1,κ	B7, B27	*†*
WAR 5D5	H	IgG1,κ	B7, B27, B42, B55	*†*
WIM 8E5	H	IgG1,κ	A1, A10, A11, A9, A29, A30, A31, A33	*†*
WK 3D10	H	IgM,κ	A2, A3, A23, A31, B7, B13, B17, B21, B40, B62	*†*
WK 4E3	H	IgM,λ	A locus (not A1, A24)	*†*

M, Mouse; H, Human; R, Rat; *, kindly donated by Dr. K. Gelsthorpe, Sheffield, U.K.; †, this paper.

**Table 2:** HLA genotype and phenotype of the 21 patients whose tumor tissue was used for flow cytometry.

case	A.1	A.2	B.1	B.2
40	A2	A3	B15	B38
43	A2	A3	B18	B49
44	A2	A24	B7	B55
45	A1	A2	B8	B62
48	A3	A3	B7	B62
55	A1	A3	B8	B62
56	A1	A30	B8	B51
58	A2	A32	B44	B60
59	A1	A2	B7	B62
61*	A2		Bw4	Bw6
63*	A2		Bw4	Bw6
69	A2	A24	B7	B39
106*	A3		Bw4	Bw6
108	A2	A68	B51	B53
109*	A3		Bw4	Bw6
110	A1	A23	B8	B50
120	A2	A3	B7	B44
122	A1	A24	B44	B56
124*	A2		Bw4	
179	A3	A3	B7	B35
191	A2	A24	B15	B40

A.1, A.2, B.1 and B.2 indicate the different HLA A and B alleles. * HLA genotype was not known prior to tumor resection, phenotype based on flow cytometry of vim+ cells with mouse monoclonal antibodies (Table 1).
