# Supplementary material for: High-resolution analysis of HLA class I alterations in colorectal cancer
Source: BMC Cancer. 2006 Oct 2;6:233. doi: 10.1186/1471-2407-6-233 (PMC1599746; doi:10.1186/1471-2407-6-233)
Supplement: Additional File 1 — HLA phenotype alterations and clinicopathological features of colorectal tumors and their mismatch repair status. These data provides the HLA phenotypes assessed by flow cytometry of the different tumors and their clinicopathological features and mismatch repair status. Clinicopathology: loc., tumor localization: A, colon ascendens; Ce, caecum; D, colon descendens; R, rectum; RS, rectosigmoid; S, sigmoid; modified Dukes stages [43]; F-U, follow-up (max. 2 years): DOD, dead of disease; DND, dead but not due to disease; M, distant metastasis; nr, no recurrences. MMR, mismatch repair status: MSI, microsatellite instability: H, MSI-High; S, microsatellite stable; *, HNPCC, hereditary non-polyposis colorectal cancer; +, tumor epithelium staining positive as described in text; -, no staining of tumor cells, but staining of 'normal' cells, - +, heterogeneous staining of tumor cells; FCM, flow cytometry: A, aneuploid; D, diploid; M, multiploid; HLA FCM: Relative HLA Expression Values were calculated from flow cytometry analyses as described in the methods section. Depicted in black are cases in which fluorescence intensity of ker+ cells was equal to the negative control (see Figure 1). A.1 – B.2, HLA-A and -B alleles as depicted in Table 2; HC, HLA heavy chain expression detected with the W6/32 antibody. †, HLA-A and -B negative and positive populations are present, therefore REV is not informative (see Figure 2); ‡, homozygous HLA-A genotype. HLA IHC, immunohistochemistry of HLA molecules: +, tumor epithelium staining positive as described in text; -, no staining of tumor cells, but staining of 'normal' cells; c, tumor epithelium staining restricted to the cytoplasm; -+, heterogeneous staining of tumor epithelium. [file 1471-2407-6-233-S1.pdf]

	clinicopathology					MMR			FCM	HLA FCM - expression values						HLA IHC		
case	age	sex	loc.	Dukes	F-U	MSI	MLH1	PMS2	ploidy	A.1	A.2	B.1	B.2	HC	B2M	HCA2	HC10	B2M
61	64	F	Ce	C2	DOD	S	+	+	A	0,06		0,70	0,14	1,86	2,41	+	- +	- +
63	57	F	Ce	B2	nr	S	+	+	D	0,16		2,17	4,30	4,51	5,59	- +	- +	- +
109	68	M	A	C2	M	S	+	+	A	1,77		0,16	1,13	1,47	1,90	- +	- +	- +
191	70	M	A	C3	nr	H	-	-	D	0,02	4,21	0,30	0,47	1,52	1,56	+	- +	+
55	74	F	Ce	B2	nr	H	-	-	M	†	†	†	†	0,15	0,35	-	-	c
56	75	M	Ce	B3	DND	H	-	-	D	0,01	0,05	0,20	0,01	0,40	0,28	-	-	c
120	62	F	S	B2	nr	H*	+	+	D	0,01	0,15	0,10	0,08	0,45	0,59	-	-	c
179	90	M	Ce	B2	nr	H	-	-	D	0,07	0,07‡	0,06	0,05	0,53	0,49	-	-	c
40	81	F	R	C3	DOD	S	+	+	A	2,37	3,05	7,27	>15	0,46	2,64	+	+	- +
43	50	M	R	C2	DOD	S	+	+	M	10,71	8,24	8,34	11,50	4,80	7,95	+	+	+
44	57	M	S	B2	nr	S	+	+	A	9,72	10,32	7,17	2,72	5,85	3,87	- +	- +	+
45	64	F	RS	A	nr	S	+	+	D	2,91	3,86	1,36	3,18	1,56	1,00	- +	- +	- +
48	66	F	S	B2	nr	S	+	+	D	5,65	5,65‡	2,75	2,74	1,29	1,76	- +	- +	- +
58	53	F	D	B2	nr	S	+	+	M	1,10	0,72	1,36	1,00	1,12	1,53	- +	- +	- +
59	56	M	R	B1	nr	S	+	+	D	2,15	2,73	1,56	1,35	1,81	2,40	- +	- +	- +
69	74	F	A	B2	M	S	+	+	A	1,87	2,34	1,28	0,90	1,90	2,20	- +	- +	- +
106	61	F	A	C2	M	S	+	+	M	1,41		0,64	0,87	1,00	1,36	+	+	- +
108	52	M	A	C2	nr	S	+	+	M	1,37	1,34	0,59	0,71	1,31	1,51	- +	- +	- +
110	74	F	S	C2	M	S	+	+	D	3,00	4,53	1,53	1,86	2,81	4,46	- +	+	- +
122	55	F	S	C2	M	S	+	+	A	1,10	2,54	1,37	1,22	1,16	1,73	- +	+	- +
124	75	F	Ce	C1	nr	S	+	+	A	5,13		1,58		2,98	3,76	- +	- +	- +
